# Factors associated with maintenance of the use of internet, *EpiFloripa Idoso* longitudinal study

**DOI:** 10.11606/S1518-8787.2018052000216

**Published:** 2018-03-14

**Authors:** Rodrigo de Rosso Krug, André Junqueira Xavier, Eleonora d’Orsi

**Affiliations:** I Universidade Federal de Santa Catarina. Programa de Pós-Graduação em Ciências Médicas. Florianópolis, SC, Brasil; II Universidade de Cruz Alta. Programa de Pós-Graduação em Atenção Integral em Saúde. Cruz Alta, RS, Brasil; III Universidade Federal de Santa Catarina. Programa de Pós-Graduação em Saúde Coletiva. Florianópolis, SC, Brasil

**Keywords:** Aged, Computer Literacy, Computers, utilization, internet, utilization, Attitude to Computers, Digital Divide, Socioeconomic Factors, Idoso, Conhecimentos em Informática, Computadores, utilização, Internet, utilização, Atitude Frente aos Computadores, Exclusão Digital, Fatores Socioeconômicos

## Abstract

**OBJECTIVE:**

To describe the use of the internet and to identify the sociodemographic and health factors associated with changes in the use of the internet over four years in older adults participating in the Brazilian *EpiFloripa Idoso* study.

**METHODS:**

This is a longitudinal home-based, population-based study with 1,197 older adults who live in the urban area of Florianópolis, State of Santa Catarina, Brazil. We applied a face-to-face interview. We describe the use of internet according to frequency, place, devices, and motives of the use of the internet. To identify factors associated with changes in the use of the internet, we categorized the outcome as: non-use of the internet, stopped using the internet, started using the internet, and kept using the internet. The independent variables were sex, age, family income, education level, family arrangement, marital status, presence of caregiver, paid work, and cognitive impairment screening. We used multinomial logistic regression with risk ratio (RR) estimates and their respective confidence intervals (95%CI).

**RESULTS:**

The prevalence of the use of internet increased from 22.9% in 2009–2010 to 26.6% in 2013–2014. Of the total number of older adults who participated in the study, 7.5% started using the internet, 3.2% stopped using it, 19.1% kept their use, and 70.2% kept their non-use in the analyzed period. Of the older adults who used the internet, most used it every day or almost every day of the week, in their own home, on desktop or portable computers, mainly to send and receive messages, to search for information to learn or investigate something, to find information about goods and services, and to use news, social networking, and health information websites. The factors associated with the use of internet over four years were: being male (RR = 2.19, 95%CI 1.48–3.26), higher monthly family income (RR = 3.53, 95%CI 1.35–9.23), higher education level (RR = 2.64, 95%CI 1.57–4.43), and no presence of caregiver (RR = 0.08, 95%CI 0.02–0.31).

**CONCLUSIONS:**

Although the use of the internet is increasing among older adults, most of the population is still digitally excluded, especially women with lower income and education level. Strategies that promote the digital inclusion of older adults should be stimulated, aiming to universalize the use of the internet, and they should take into account socioeconomic and gender inequalities.

## RESUMO

**OBJETIVO:**

: Descrever o uso de internet e identificar os fatores sociodemográficos e de saúde associados a mudanças no uso de internet ao longo de quatro anos em idosos participantes do estudo EpiFloripa Idoso.

**MÉTODOS:**

: Estudo longitudinal de base populacional e domiciliar com 1.197 idosos residentes na área urbana de Florianópolis, SC. Aplicou-se entrevista face a face. A descrição do uso de internet foi realizada segundo a frequêcia, o local, os aparelhos e os motivos de uso da internet. Para identificar os fatores associados a mudanças no uso de internet, o desfecho foi categorizado em: manteve o não uso de internet; passou a não usar internet; passou a usar internet; e manteve o uso de internet. As variáveis independentes foram sexo, idade, renda familiar, escolaridade, arranjo familiar, *status* conjugal, presença de cuidador, trabalho remunerado e rastreamento de déficit cognitivo. Utilizou-se regressão multinomial logística com estimativas de razão risco relativo (RRR) e seus respectivos intervalos de confiança (IC95%).

**RESULTADOS:**

: A prevalência de uso de internet aumentou de 22,9% em 2009–2010 para 26,6% em 2013–2014. Do total de idosos participantes do estudo, 7,5% passaram a usar internet, 3,2% deixaram de usar, 19,1% mantiveram o uso e 70,2% mantiveram o não uso no período analisado. Dos idosos que usavam internet, a maioria usava todos os dias ou quase todos os dias da semana, em sua própria casa, em computadores de mesa ou portáteis, com o principal objetivo de enviar e receber mensagens, buscar informações para aprender ou investigar alguma coisa, encontrar informações sobre bens e serviços e usar sites de notícias, de redes sociais e de informações sobre saúde. Os fatores associados à manutenção, ao longo de quatro anos, do uso da internet foram: ser do sexo masculino (RRR = 2,19; IC95% 1,48–3,26), ter maior renda salarial mensal familiar (RRR = 3,53; IC95% 1,35–9,23), ter maior escolaridade (RRR = 2,64; IC95% 1,57–4,43) e não ter cuidador (RRR = 0,08; IC95% 0,02–0,31).

**CONCLUSÕES:**

: Apesar de o uso da internet estar aumentando entre os idosos, a maior parte dessa população ainda é digitalmente excluída, especialmente as mulheres de menor renda e escolaridade. Estratégias que promovam a inclusão digital de idosos devem ser estimuladas visando à universalização do uso de internet e devem levar em consideração as desigualdades socioeconômicas e de gênero.

## INTRODUCTION

The internet is the largest global information system formed by a worldwide network of interconnected computers^1^. Since its creation, the internet has rapidly taken on world proportions and has been used by millions of persons, becoming central to postmodern civilization^2^.

However, the internet has intensified the process called “digital exclusion”, that is, excluding those who do not use this technology. Generally, the most digitally excluded persons are those with the worst financial conditions, those with lower education level, and older adults^3^ ^,^ ^4^.

With the increasing number of older adults around the world, the use of the internet by this group age must be increasingly considered^5^ so that they can enjoy all the benefits of this technological behavior. Older adults have been searching for computerization^6^; nevertheless, many still do not use the internet^7^. A research carried out in 2011 showed that 85% of the Brazilian older adults did not use the internet^8^, which decreased to 81% in 2014^3^.

The use of the internet by older adults, in addition to providing daily benefits, can provide knowledge, social inclusion, leisure, employment^9–11^, greater communication with family and friends, strengthening of intergenerational relations^3^, health benefits^11^, reduced number of diseases, improved autonomy and quality of life^12^ ^,^ ^13^, reduced risk of impairment of the activities of daily living^14^, and reduced physical, mental, and socioeconomic limitations associated with aging^11^.

The presence of this technological behavior is one of the most effective ways to keep or improve the cognitive function^12^ ^,^ ^13^ ^,^ ^15^ ^,^ ^16^, which can improve creativity, knowledge acquisition, attention, execution of tasks, and other cognitive abilities^17^.

However, even with all the benefits that the use of the internet provides, there are still many inequalities in the access to this technology. A significant portion of the population is still digitally excluded for a variety of reasons, such as the cost of access, lack of places to access, lack of knowledge about the internet, among many other factors that worsen with increasing age^3^ ^,^ ^7^ ^,^ ^18^.

Thus, the knowledge about the profile of use of the internet by older adults^4^ ^,^ ^6^ ^,^ ^19^ and which factors may interfere in this use may help in the development of actions that stimulate the use of this tool by older adults, contributing to keeping or improving the cognitive performance, social participation, quality of life, and health of users.

Some studies^6,7,19–21^ have researched this subject, but none has done so with a representative sample of a city and longitudinally. Thus, the objective of this study was to describe the use of the internet and to identify the sociodemographic and health factors associated with changes in the use of the internet over a four-year period in older adults.

## METHODS

Here, we report the results of the longitudinal home-based, population-based study named *EpiFloripa Idoso*, with baseline in 2009-2010 and follow-up in 2013–2014.

This study was carried out with older adults (aged 60 years and over) living in the urban area of Florianópolis, State of Santa Catarina, Brazil. The sample of this study originated initially in the baseline of the research, from September 2009 to June 2010 (2009–2010 *EpiFloripa Idoso*).

The baseline sample size was calculated using the EpiInfo Version 6.04 Program, based on the population size of the municipality (44,460 older adults), the confidence level (95%), the unknown prevalence of the phenomenon (50%), the sampling error (four percentage points), the sampling design (estimated as two), plus 20% for estimated losses and 15% for association studies, resulting in a sample of 1,599 individuals. The sample was selected by conglomerates, in two stages (first stage: 420 urban census tracts of Florianópolis, State of Santa Catarina, Brazil – 2000 Census –, being 80 tracts drawn systematically, that is, eight tracts for each income decile; second stage: households). Given the availability of financial resources, it was estimated that 23 interviews should be carried out per census tract, allowing greater variability of the sample, thus amounting to 1,911 eligible individuals for the study. The final sample consisted of 1,705 older adults who were effectively interviewed (response rate of 89.1%).

In the follow-up of the study from November 2013 to November 2014 (2013–2014 *EpiFloripa*), the sample was obtained from the identification of the deaths present in the database of the Mortality Information System of 2009, 2010, 2011, 2012, and 2013 of the Ministry of Health. Subsequently, letters were sent to the older adults who had full address and a telephone contact was carried out to update the recorded data. When communication was not possible, the team sought to update these data using the *InfoSaúde* System (Health System of Florianópolis), social networks, phone book, and contact with neighbors, relatives, and friends. An individual under 60 years of age who was mistakenly interviewed at the baseline and an older adult erroneously recorded twice in the database were also excluded from the study.

The institutionalized older adults (nursing homes, hospitals, prisons) were excluded. The losses corresponded to older adults not located after four visits in different periods and refusals when expressed personally after home visit and attempted interview. In the follow-up, the older adults who changed cities or were hospitalized were also considered as losses. Thus, 1,197 older adults were interviewed (follow-up ratio of 70.2%).

A structured questionnaire with 276 questions was used in the baseline and a structured questionnaire with 655 questions was used in the follow-up. Validated instruments were preferably used, and all were tested in a pilot study (n = 76 older adults). Data consistency was checked weekly and quality control was performed with the application of a reduced questionnaire in approximately 10% of the interviewees, randomly selected, using the telephone. The reproducibility of the questions presented an agreement between satisfactory and good (kappa between 0.5 and 0.9). Data collection was performed with a face-to-face interview applied by previously trained interviewers with the assistance of a palm top (2009–2010) and netbook (2013–2014). The complete tools are available at www.epifloripa.ufsc.br and more information on the *EpiFloripa Idoso* study can be found in the study of Confortin et al.^22^ To describe the use of the internet, we used the variables of the questionnaire applied in 2013-2014 containing the following items: (i) Use of internet (no, yes); (ii) How often do you use the internet or email (every day or almost every day, at least once a week, at least once a month, at least once every three months, less than every three months); (iii) Where do you use it (at home, at work, at a place of study, at someone else’s home, other places); (iv) In which devices do you use it (desktop or portable computer, tablet or smartphone, others); and (v) For what purpose do you use it (to send and receive messages, to find information about goods and services, to search for information to learn or investigate something, for finance, online shopping, sales of goods or services, social networking websites, to upload or share content, for news websites, to watch TV channels and listen to the radio, for games, to look for work or send a job application, for information about health, information about healthy eating, information about physical activity, other)^23^.

In order to identify the sociodemographic and health factors associated with changes in the use of internet over the four-year period, the outcome variable was the use of the internet, which was longitudinally evaluated (kept the non-use of the internet, stopped using the internet, started using the internet, kept using the internet), and the independent variables were:

- Sociodemographic aspects: sex (female, male), age (in full years), per capita family income in minimum wages (MW) at the time of the interview (≤ 1 MW, 1.1–3 MW, 3.1–5 MW, 5.1–10 MW, > 10 MW), education level (0–4 years, 5–8 years, 9 years or more), family arrangement (lives with companion, lives alone), marital status (married, single, divorced, widowed), presence of caregiver (no, yes), and paid work at the time of the interview (no, yes);
- Health aspects: evaluated by screening for cognitive impairment using the Mini-Mental State Examination (MMSE), a cognitive assessment scale validated in Brazil ranging from zero to 30 points^24^. Its classification is given by the education level; older adults are considered to have a likely cognitive impairment if they reach values lower than 19/20 points (older adults with no schooling) and values lower than 23/24 points (older adults with formal education)^25^. Trentini et al.^26^ explain that cognitive impairment has a strong relation with the worsening of the health of older adults.

The interviews were downloaded to the netbook in the csv format and subsequently exported to the statistical package Stata 11.0 (StataCorp. 2009. Stata Statistical Software: Release 11, College Station, TX: StataCorp LP), which eliminates the typing step, thus reducing the possible errors that occur during this step.

We considered the design effect and the sample weights in all the analyses related to this study. We evaluated the normality of the data using the Kolmogorov-Smirnov test. To describe the profile of use of the internet, we performed descriptive statistics using absolute and relative frequencies and their respective 95% confidence intervals (95%CI).

To identify the factors associated with keeping the use of internet after the four-year period in the older adults participating in the 2013–2014 *EpiFloripa Idoso* study, we used the crude analysis and the analysis adjusted for multinomial logistic regression with risk ratio (RR) estimates and respective 95%CI. In the adjusted analysis, the variables that showed association with the outcome (p ≤ 0.05) were inserted into the final model.

The research project complied with ethical precepts, according to Resolution 466 of 2012, of the National Health Council, being approved by the Research Ethics Committee of the Universidade Federal de Santa Catarina (baseline under Protocol 352/2008 and Follow-up 596,126). All interviewees signed the informed consent.

## RESULTS

The prevalence of use of the internet among older adults in the 2013–2014 EpiFloripa study was 26.6% (95%CI 24.1–29.1) (Table 1). Of them, 7.5% (95%CI 6.0–9.0) started using the internet in the four-year period, 3.2% (95%CI 2.2–4.2) stopped using the internet, and 19.1% (95%CI 16.8–21.3) kept using it. However, we noticed a high prevalence of the non-use of the internet (70.2%, 95%CI 67.6–72.8). When comparing the prevalence in the two moments of the study (22.9% in 2009–2010 and 26.6% in 2013–2014), we verified that the older adults participating in the *EpiFloripa Idoso* study increased the use of the internet in the follow-up.

**Table 1:** Description of the profile of use of internet of the participants of the 2013–2014 *EpiFloripa Idoso* study. Florianópolis, State of Santa Catarina, Brazil, 2014.

Variable	n	%	95%CI
Use of the internet			
Kept the non-use of the internet	839	70.2	67.6–72.8
Stopped using the internet	38	3.2	2.2–4.2
Started using the internet	90	7.5	6.0–9.0
Kept using the internet	228	19.1	16.8–21.3
How many times do you use the internet			
Every day or almost every day	254	79.9	75.4–84.3
At least once a week	53	16.7	12.5–20.8
At least once a month	6	1.9	0.4–3.4
At least once every three months	4	1.3	0.0–2.5
Less than every three months	1	0.3	-0.3–0.9
Where do you use it*			
At home	305	95.9	93.7–98.1
At work	43	13.5	9.7–17.3
At a place of study	10	3.1	1.2–5.1
At someone else’s home	25	7.9	4.9–10.8
At other places	15	4.7	2.4–7.1
In which devices do you use it*			
Desktop or portable computer	303	95.3	92.9–97.6
Tablet or smartphone	87	27.4	22.4–32.3
Other	2	0.6	-0.2–1.5
What do you use it for*			
To send or receive messages	272	85.5	81.6–89.4
To find information about goods and services	216	67.9	62.8–73.1
To search for information to learn or investigate something	249	78.3	73.7–82.9
Finance	75	23.6	18.9–28.3
Online shopping	101	31.8	26.6–36.9
To sell goods or services	9	2.8	1.0–4.7
To use social networking websites	207	65.1	59.8–70.4
To create, upload, or share content	76	23.9	19.2–28.6
News websites	208	65.4	60.2–70.7
To watch TV channels and to listen to the radio	64	20.1	15.7–24.6
Games	105	33.0	27.8–38.2
To look for a job or to send a job application	1	0.3	-0.3–0.9
Information about health	204	64.2	58.8–69.5
Information about a healthy diet	180	56.6	51.2–62.1
Information about physical activity	95	29.9	24.8–34.9
Other	32	10.1	6.7–13.4

* Questions which could have more than one answer.

Most of the older adults who used the internet did it so every day or almost every day of the week, in their own home, on desktop or portable computers. These older adults had as their main goal the use of the internet to send and receive messages, to seek information to learn or investigate something, to find information about goods and services, and to use news, social networking, and health information websites (Table 1).

Table 2 shows the distribution of the percentage of the sociodemographic and cognitive characteristics of the older adults according to keeping the non-use of the internet, stopping using the internet, starting using the internet, and keeping using the internet after the four-year period. Most of the older adults that kept using the internet between baseline and follow-up had family income above 10 minimum wages, nine or more years of education, and lived alone. In addition, the prevalence of use was higher in males and among older adults who lived alone, had no caregiver, had paid work, and had no cognitive impairment.

**Table 2:** Distribution of the percentage of sociodemographic and cognitive characteristics according to the variables of ‘kept the non-use of the internet’, ‘stopped using the internet’, ‘started using the internet’, and ‘kept the use of internet’ after the four-year period of the participants of the *EpiFloripa Idoso* study. Florianópolis, State of Santa Catarina, Brazil, 2014.

Variable	Kept the non-use of the internet	Stopped using the internet	Started using the internet	Kept the use of the internet
			
	% (95%CI)	% (95%CI)	% (95%CI)	% (95%CI)
Sex				
Female	72.5 (68.2–76.9)	2.7 (1.3–4.0)	8.4 (5.8–11.3)	16.4 (12.3–20.4)
Male	58.9 (50.6–67.3)	4.6 (2.2–7.1)	7.7 (4.5–10.9)	28.7 (21.5–35.9)
Family income (minimum wage)				
≤ 1	90.4 (85.0–95.7)	1.4 (-0.02–3.1)	2.4 (-0.4–5.3)	6.2 (1.7–9.8)
From 1 to 3	84.9 (80.4–89.4)	3.4 (0.7–6.0)	5.5 (2.3–8.7)	6.2 (2.9–9.4)
From 3 to 5	73.4 (63.3–83.5)	2.7 (0.6–4.9)	7.9 (3.8–12.1)	15.9 (6.9–24.8)
From 5 to 10	63.2 (55.2–71.2)	2.7 (0.7–4.7)	10.4 (5.6–15.2)	23.6 (15.5–31.8)
> 10	31.6 (25.6–37.6)	5.9 (1.0–10.7)	5.8 (17.0–9.8)	50.9 (41.6–60.2)
Education level (years)				
0–4	64.9 (60.2–69.7)	17.5 (12.9–22.8)	8.7 (5.6–11.8)	8.8 (5.7–11.9)
5–8	62.9 (54.9–70.9)	17.2 (11.9–22.5)	10.7 (6.0–15.3)	9.2 (4.8–13.5)
9 or more	45.2 (37.0–53.4)	15.8 (9.7–21.9)	13.7 (9.8–17.6)	25.2 (19.3–31.5)
Family arrangement				
With companion	68.3 (63.0–73.5)	3.2 (1.8–4.6)	8.0 (6.0–10.0)	20.5 (15.9–25.1)
Alone	64.8 (57.3–72.2)	4.0 (0.8–7.2)	8.8 (4.1–13.5)	22.4 (15.0–29.8)
Marital status				
Married	63.6 (57.4–69.8)	4.2 (2.3–6.0)	74.9 (5.3–9.7)	24.7 (18.7–30.7)
Single	63.2 (46.9–79.5)	1.8 (-0.8–4.5)	3.6 (0.0–7.2)	31.4 (15.1–47.6)
Divorced	58.5 (46.6–70.4)	1.5 (-0.1–4.5)	9.9 (1.9–17.8)	30.0 (17.9–42.1)
Widow	77.7 (72.8–8.5)	2.7 (0.6–4.9)	9.8 (5.7–13.9)	9.8 (6.1–13.6)
Presence of caregiver				
No	65.1 (60.1–70.2)	3.2 (1.8–4.5)	8.6 (6.6–10.7)	23.1 (18.2–28.0)
Yes	90.2 (82.7–97.7)	5.4 (-0.2–11.1)	3.8 (-2.2–9.8)	0.5 (-0.5–1.5)
Paid work				
No	69.6 (64.9–74.2)	3.7 (1.9–5.3)	8.2 (6.2–10.2)	18.5 (14.0–23.0)
Yes	47.1 (32.2–62.1)	2.5 (-0.1–5.0)	10.8 (3.2–18.4)	39.5 (26.4–52.7)
Cognitive impairment				
No	58.4 (53.0–63.8)	3.7 (1.9–5.5)	10.1 (7.7–12.5)	27.8 (22.6–32.9)
Likely impairment	94.0 (90.9–97.1)	2.6 (0.7–4.4)	2.7 (0.8–4.6)	0.7 (-0.4–1.8)

RR: risk ratio

In the crude analysis (Table 3), older adults who kept using the internet in the four-year period was associated with males, younger age, higher education level, not being a widower, no presence of a caregiver, and no cognitive impairment when compared to females, older age, being a widower, presence of a caregiver, and presence of cognitive impairment, respectively.

**Table 3:** Crude analysis of the factors associated with the variables of ‘stopped using the internet’, ‘started using the internet’, and ‘kept using the internet’ after the four-year period of the participants of the *EpiFloripa Idoso* study. Florianópolis, State of Santa Catarina, Brazil, 2014.

Variable	Stopped using the internet		Started using the internet		Kept using the internet	
		
	RR (95%CI)	p	RR (95%CI)	p	RR (95%CI)	p
Sex		0.152		0.376		< 0.001
Female	1		1		1	
Male	1.62 (0.84–3.13)		1.23 (0.78–1.94)		2.07 (1.54–2.80)	
Age	0.95 (0.91–1.00)	0.053	0.95 (0.91–0.98)	0.001	0.90 (0.88–0.92)	< 0.001
Family income (minimum wage)		0.004		< 0.001		< 0.001
≤ 1	1		1		1	
From 1 to 3	1.31 (0.35–4.92)		2.33 (0.67–8.14)		0.87 (0.38–2.00)	
From 3 to 5	1.72 (0.44–6.81)		4.93 (1.43–16.96)		2.38 (1.10–5.21)	
From 5 to 10	2.29 (0.62–8.49)		5.49 (1.62–18.64)		4.43 (2.12–9.26)	
> 10	5.28 (1.38–20.14)		15.26 (4.46–52.20)		22.30 (10.65–46.67)	
Education level (years)		0.342		0.004		< 0.001
0–4	1		1		1	
5–8	1.01 (0.66–1.56)		1.27 (0.65–2.48)		1.07 (0.55–2.88)	
9 or more	1.30 (0.76–2.23)		2.26 (1.31–3.88)		4.09 (2.48–6.75)	
Family arrangement		0.776		0.945		0.075
With companion	1		1		1	
Alone	1.12 (0.50–2.50)		0.98 (0.56–1.71)		1.37 (0.97–1.94)	
Marital status		0.072		0.136		< 0.001
Married	1		1		1	
Single	0.66 (0.15–2.88)		0.66 (0.23–1.91)		1.17 (0.67–2.03)	
Divorced	0.30 (0.04–2.26)		1.36 (0.63–2.90)		1.17 (0.69–1.99)	
Widow	0.49 (0.23–1.06)		0.68 (0.41–1.13)		0.38 (0.26–0.55)	
Presence of caregiver		0.616		0.008		< 0.001
No	1		1		1	
Yes	0.76 (0.26–2.19)		0.15 (0.03–0.61)		0.03 (0.01–0.20)	
Paid work		0.859		0.113		0.310
No	1		1		1	
Yes	1.00 (1.00–1.00)		1.00 (1.00–1.00)		1.00 (1.00–1.00)	
Cognitive impairment		0.080		< 0.001		< 0.001
No	1		1		1	
Likely impairment	0.49 (0.22–1.09)		0.18 (0.09–0.38)		0.06 (0.01–0.07)	

RR: risk ratio

In the adjusted analysis (Table 4), the older adults who kept using the internet in the four-year period (2009–2010 to 2013–2014) were associated with sex – men were approximately twice as likely (RR = 2.19, 95%CI 1.48–3.26) to keep using the internet when compared to women; monthly family income and education level – the older adults who received more than 10 MW (RR = 3.53, 95%CI 1.35–9.23) and who had more than nine years of education (RR = 2.64, 95%CI 1.57–4.43) were more likely to keep using the internet (approximately two to three times, respectively) when compared to those with lower income and education level; and the absence of caregiver (RR = 0.08, 95%CI 0.02–0.31) – as older adults with caregivers had almost 90% less chance of having this technological behavior when compared to older adults without a caregiver.

**Table 4:** Adjusted analysis of the factors associated with the variables of ‘stopped using the internet’, ‘started using the internet’, and ‘kept using the internet’ after the four-year period of the participants of the *EpiFloripa Idoso* study. Florianópolis, State of Santa Catarina, Brazil, 2014.

Variable	Stopped using the internet		Started using the internet		Kept using the internet	
		
	RR (95%CI)	p	RR (95%CI)	p	RR (95%CI)	p
Sex		0.341		0.305		< 0.001
Female	1		1		1	
Male	1.27 (0.77–2.07)		1.30 (0.78–2.18)		2.19 (1.48–3.26)	
Age	1.00 (0.98–1.03)	0.606	0.97 (0.94–1.00)	0.069	0.98 (0.94–1.03)	0.436
Family income (minimum wage)		0.576		0.389		
≤ 1	1		1		1	0.018
From 1 to 3	0.75 (0.43–1.30)		0.57 (0.26–1.25)		2.34 (1.05–5.21)	
From 3 to 5	0.52 (0.24–1.11)		1.38 (0.68–2.83)		1.39 (0.51–3.78)	
From 5 to 10	0.63 (0.32–1.23)		1.03 (0.44–2.39)		2.51 (1.02–6.17)	
> 10	0.84 (0.40–1.76)		1.08 (0.43–2.69)		3.53 (1.35–9.23)	
Education level (years)		0.450		0.031		< 0.001
0–4	1		1		1	
5–8	0.99 (0.64–1.52)		0.94 (0.59–1.50)		0.98 (0.48–2.01)	
9 or more	1.23 (0.73–2.06)		1.18 (0.63–2.21)		2.64 (1.57–4.43)	
Family arrangement		0.722		0.078		0.052
With companion	1		1		1	
Alone	1.07 (0.72–1.61)		1.56 (0.95–2.55)		1.58 (0.98–2.55)	
Marital status		0.978		0.516		0.181
Married	1		1		1	
Single	1.25 (0.41–3.77)		0.97 (0.41–2.32)		1.07 (0.47–2.47)	
Divorced	0.68 (0.31–1.49)		0.42 (0.19–0.93)		0.46 (0.21–1.03)	
Widow	1.07 (0.49–2.32)		0.69 (0.37–1.30)		0.75 (0.42–1.32)	
Presence of caregiver		0.001		0.462		< 0.001
No	1		1		1	
Yes	0.32 (0.16–1.64)		0.68 (0.24–1.90)		0.08 (0.02–0.31)	
Paid work		0.054		0.512		0.780
No	1		1		1	
Yes	1.00 (1.00–1.00)		1.00 (1.00–1.00)		1.00 (1.00–1.00)	
Cognitive impairment		0.821		0.006		0.062
No	1		1		1	
Likely @impairment	1.05 (0.65–1.71)		0.53 (0.34–0.83)		0.64 (0.40–1.02)	

RR: risk ratio

## DISCUSSION

The prevalence of the use of the internet among older adults participating in the *EpiFloripa Idoso* study increased during the study period, going from 22.9% in the baseline to 26.6% in the follow-up. This increase in the use of the internet can be explained by the fact that older adults are now seeking to enter the virtual world^6^ and the current lower cost to access the internet^7^ ^,^ ^20^. In addition, the prevalence of the use of the internet of 26.6% can be considered expressive when compared with the Brazilian (19.0%)^3^ and Portuguese prevalence (20.9%)^27^.

Among the researched older adults who used the internet, most used this technology every day or almost every day of the week. The study of Dias^19^, performed with 30 Portuguese older adults (55 to 90 years old) from different municipalities has also shown that most of the older adults who use the internet often use it. Data from Cetic^3^ show that most of the older adults who use the internet have this behavior on a daily basis. Kachar^20^ reports that internet access by older adults is small, but those with such behavior use it as often as other age groups.

Another finding in our study is that the older adults studied used the internet in their own home on desktop or portable computers. Other studies^3^ ^,^ ^6^ ^,^ ^19^ ^,^ ^27^ have also found similar findings. The use of the internet at home is increasing because of the decreasing financial cost for the acquisition of this technology^3^ ^,^ ^7^ ^,^ ^20^ and the convenience of using it at home^27^.

Regarding the main goal for the use of the internet, most of the older adults reported using it to send and receive messages, to seek information to learn or investigate something, to find information about goods and services, to use news websites, to use social networking websites, and to search information about health. Verona et al.^21^, when researching 32 individuals (mean age = 68.8 years; SD = 6.9 years) from three institutions for older adults in the city of São Paulo, Brazil, have found similar data: of those who use the internet, 13.5% used to search for something, 10.2% for news, 6.8% for services in general, and 3.4% for communication with other persons.

The use of the internet to send and receive messages and to use social networks is very common for the older adults who frequently use this technology^7^ ^,^ ^19^. This may be because the internet is a very interesting and fast tool for communicating with family and friends who are distant^19^. It may also be because most older adults are socially excluded, making them seek the internet as a means to increase their participation in communication and socialization networks^6^ ^,^ ^11^ ^,^ ^21^. According to data from the research of Cetic^3^, the use of social networks and e-mail is frequent in older adults who use the internet (70% and 77%, respectively).

The search for information to learn or investigate something, to find goods and services, to research about health, and to read the news are also one of the main reasons that lead older adults to use the internet^6^ ^,^ ^19^. This can be explained by the convenience and ease that the internet can provide to vulnerable persons, given the difficulty that older adults may encounter to leave their homes in search for such information and services. In this sense, the internet provides access to different and useful information for these persons^19^ ^,^ ^20^.

The use of the internet to search for information about health, besides being frequent in older adults, shows that this technological tool has an enormous potential to inform and facilitate the changes of behavior in relation to the health of this population^4^. In addition, the use of the internet by older adults can provide several health benefits, such as: social inclusion^9–11^, improved autonomy and quality of life^12^ ^,^ ^13^, and reduced risk of impairment of the activities of daily living^14^, the limitations from aging^11^, and the number of diseases.

In relation to the association of the use of the internet with the sociodemographic and health variables of the older adults participating in the 2013–2014 *EpiFloripa Idoso* study, males were twice as likely (RR = 2.19, 95%CI 1.48–3.26) to keep using the internet when compared to women. This difference between sexes corroborates the study of Simões et al.^28^ In addition, men are more dependent on the internet in all aspects, whether for work, for communication, or for leisure^29^.

Monthly family income and education level were also associated with keeping using the internet in the older adults in our study. Older adults who received more than 10 minimum wages (RR = 3.53, 95%CI 1.35–9.23) and had more than nine years of education level (RR = 2.64, 95%CI 1.57–4.43) were more likely to have this behavior when compared to their peers.

Monthly income is an important factor for access to education level and the internet^7^ ^,^ ^18^. Castells^30^ explains that older adults often cannot afford to own a computer or have access to the internet at home because they generally only receive their retirement pension. Education level is also directly related to the lower use of the internet by older adults, in which subjects with lower education levels may have more difficulty using and manipulating the computer and the internet^19^. In addition, the proportion of internet users increases among the population with higher education level^3^ ^,^ ^18^.

When low income, low education level, and increasing age are combined, the use of the internet is even more difficult, as these older adults need to be convinced that the digital world is possible, both in the financial and educational context. In this sense, public policies should be stimulated for the universalization of the access to the internet by older adults with low income and education level^7^. These proposals should aim to reduce the costs of the internet and the devices needed to use it (computers, tablets, smartphones, among others), and an alternative would be to unlink the internet from other services such as cable television and telephone lines. In addition, courses should also be considered to help older adults learn how to use and get to know the internet better^4^.

Lincoln et al.^31^ explain that low education level and low income are strong influencers of health problems such as low learning, loss of independence and functionality, and worsening of the quality of life.

The presence of a caregiver is also a factor associated with the use of internet in the older adults researched. The older adults without caregivers had greater chances of using the internet when compared to those who had a caregiver. This is probably because older adults who have caregivers are more likely to be dependent and incapacitated^32^, to have functional and cognitive problems, and, consequently, to have worse health^21^.

Moreover, Seima et al.^33^ explain that caregivers usually do not use some technologies such as smartphones, radios, televisions, computers, and the internet to dedicate themselves exclusively to the care of the older adult; with this, this caregiver ends up not influencing and not helping the older adult in the use of technologies. Given this, training courses for caregivers could explain the potential of using the internet and other technologies as possibilities for treatment and prevention of various problems arising from human aging.

The main limitations of the study were the recall bias, since the older adults may forget information, or even not know it, and the selection bias, given the non-evaluation of the older adults who were not located, the deaths, and the refusals. We suggest that qualitative research should be conducted to identify the causes that lead older adults to stop using the internet and what may influence these persons to start or keep this behavior. In addition, we suggest the development of cohort and population research studies that focus on other health variables that may influence the use of the internet by older adults. Among the positive aspects of this investigation, we highlight the design, the sample calculation, and the low proportion of selective segment loss (less than 10%).

Thus, we verified in this study that the prevalence of the use of the internet by older adults in the 2013–2014 *EpiFloripa* study was high when compared to the proportion of Brazilian older adults. We could also see that, of the older adults who used the internet, most used it every day or almost every day of the week, in their own home, on desktop or portable computers, in order to send and receive messages, search for information to learn or investigate something, find information about goods and services, and use news, social networking, and health information websites. Regarding the main finding of the study, the older adults who were more likely to use the internet in the four-year period were male, had a higher monthly family income and higher education, and had no caregiver.

The data of this research can help governments, organizations, companies, private sector, and academic institutions to elaborate and monitor public policies of digital inclusion, besides aiding in the scientific knowledge on the subject, allowing the clearer understanding of the debate around the subject of digital inclusion of older adults. In addition, the use of the internet can promote the digital inclusion of older adults also helping in the use of important tools such as e-mail, news websites, banking tasks, among many others that can facilitate the daily life of older adults. Although the use of the internet is increasing among older adults, most of the population is still digitally excluded, especially women with lower income and education level. Strategies that promote the digital inclusion of older adults should be stimulated, aiming to universalize the use of the internet, and they should take into account socioeconomic and gender inequalities.
